# Supplementary material for: A collaborative realist review of remote measurement technologies for depression in young people
Source: Nat Hum Behav. 2024 Jan 15;8(3):480–92. doi: 10.1038/s41562-023-01793-5 (PMC10963268; doi:10.1038/s41562-023-01793-5)
Supplement: Supplementary file 1 — Supplementary Tables 1–4. [file 41562_2023_1793_MOESM1_ESM.pdf]

nature human behaviour

Article

https://doi.org/10.1038/s41562-023-01793-5

A collaborative realist review of remote measurement technologies for depression in young people

In the format provided by the authors and unedited

Supplementary Table 1 - RAMESES Publication Standards: Checklist of items to be included when reporting a realist synthesis			
**Title**			
1		In the title, identify the document as a realist synthesis or review	✓
**Abstract**			
2		While acknowledging publication requirements and house style, abstracts should ideally contain brief details of: the study's background, review question or objectives; search strategy; methods of selection, appraisal, analysis and synthesis of sources; main results; and implications for practice.	✓
**Introduction**			
3	Rationale for review	Explain why the review is needed and what it is likely to contribute to existing understanding of the topic area.	✓ p.3
4	Objectives & focus of review	State the objective(s) of the review and/or the review question(s). Define and provide a rationale for the focus of the review.	✓ p.3
**Methods**			
5	Changes in the review process	Any changes made to the review process that was initially planned should be briefly described and justified.	✓ p.5
6	Rationale for using realist synthesis	Explain why realist synthesis was considered the most appropriate method to use.	✓ p.3
7	Scoping the literature	Describe and justify the initial process of exploratory scoping of the literature.	✓ p.4
8	Searching processes	While considering specific requirements of the journal or other publication outlet, state and provide a rationale for how the iterative searching was done. Provide details on all the sources accessed for information in the review. Where searching in electronic databases has taken place, the details should include, for example, name of database, search terms, dates of coverage and date last searched. If individuals familiar with the relevant literature and/or topic area were contacted, indicate how they were identified and selected.	✓ p.4 – 5 & Supplementary Table 3
9	Selection & appraisal of documents	Explain how judgements were made about including and excluding data from documents, and justify these.	✓ p.5
10	Data extraction	Describe and explain which data or information were extracted from the included documents and justify this selection.	✓ p.6
11	Analysis & synthesis processes	Describe the analysis and synthesis processes in detail. This section should include information on the constructs analysed and describe the analytic process.	✓ p.6 & Supplementary Table 4

Results			
12	Document flow diagram	Provide details on the number of documents assessed for eligibility and included in the review with reasons for exclusion at each stage as well as an indication of their source of origin (for example, from searching databases, reference lists and so on). You may consider using the example templates (which are likely to need modification to suit the data) that are provided.	✓ p.7
13	Document characteristics	Provide information on the characteristics of the documents included in the review.	✓ p.6 & Table 1
14	Main findings	Present the key findings with a specific focus on theory building and testing.	✓ p.7 - 16
**Discussion**			
15	Summary of findings	Summarize the main findings, taking into account the review's objective(s), research question(s), focus and intended audience(s).	✓ p.17
16	Strengths, limitations & future research directions	Discuss both the strengths of the review and its limitations. These should include (but need not be restricted to) (a) consideration of all the steps in the review process and (b) comment on the overall strength of evidence supporting the explanatory insights which emerged.<br>The limitations identified may point to areas where further work is needed.	✓ p.18
17	Comparison with existing literature	Where applicable, compare and contrast the review's findings with the existing literature (for example, other reviews) on the same topic.	✓
18	Conclusion & recommendations	List the main implications of the findings and place these in the context of other relevant literature. If appropriate, offer recommendations for policy and practice.	✓ p.17 - 18
19	Funding	Provide details of funding source (if any) for the review, the role played by the funder (if any) and any conflicts of interests of the reviewers.	✓ p.29

Supplementary Table 2 – Completing the Feedback Circle

Area of Project	What was planned?	What was done differently to plan / not included from YPAG feedback?	What was included / changed based on YPAG feedback?
**Proposal**	• Review of proposal / project summary, YPAG meeting with short presentation and Q&A.	• Suggestion for social media posts as a source of evidence – this was included in the submitted and funded proposal; however, in the time between the proposal and starting the project research was published that generated controversy around data mining ethics.	• Scope of the review – inclusion of RMT with both active and passive data collection; inclusion of all potential uses of RMT (symptom management, relapse-prevention & personalisation).

			• Definition of depression.<br>• 2 rather than just 1 lived-experience co-researchers.
**Lived-experience, YP co-researchers**	5 meetings:<br>• X3 training & discussion<br>• X1 discussion of results<br>• X1 discussion of feedback from YPAG<br>• Involvement in presentation of results to YPAG & leading Q&A session.<br>• Involvement in full screening & data extraction process.<br>• Co-production of deliverables.	4 meetings:<br>• X3 training & discussion<br>• X1 discussion of feedback from YPAG<br>• Literature was grouped into categories & co-researchers chose a specific area of interest for screening & data extraction.<br>• Review of deliverables.	See below for further details
**Realist review – clarifying scope**	• Discussion of initial theories of the ways, for whom and the contexts in which RMT will / won't work & review of final framework.	N/A	• Use of tech often not allowed in schools, the motivation required for RMT, digital literacy / accessibility, intrusiveness of notifications & concerns over data privacy included as theories influencing whether RMT will / won't work.
**Realist review – search strategy**	• Review of terms to be used when searching electronic databases & grey literature, anything missed?	N/A	• Inclusion of "major depressive disorder", "teenagers", "school-aged".<br>• Decision to remove "internalising disorder", "internalising symptoms" & "pre-adolescence" – childhood depression outside of 14-24 age range.
**Realist review – literature screening & selection**	• Review of inclusion & exclusion criteria.	• Inclusion of literature across different types of depression (i.e., major, bipolar, psychotic, postpartum) but out of scope for funder and constrained by time.	• Inclusion of literature where depression is the primary condition but can be other mental and/or physical co-morbidities that may influence accessibility & use of RMT.

**Realist review – data extraction**	• Inter-rater reliability testing / practice paper then data extraction in own time.	• Normally literature divided across researchers & then grouped into categories but grouped first then only literature on specific area of interest sent to co-researchers. Would have liked much more time on this.	• GN – acceptability & feasibility of RMT.<br>• TS – use of RMT in schools, concerns – data privacy, potential harms, digital divide etc.
**Results**	• YPAG meeting with short presentation and Q&A.<br>• Meeting with co-researchers to discuss results, outstanding questions & plans for YPAG meeting.<br>• Circulation of final report / results summary & outstanding questions before YPAG meeting.<br>• Involvement of co-researchers in presenting to YPAG & leading Q&A session.<br>• Meeting with co-researchers to discuss YPAG feedback.	Delay in obtaining results & writing report =<br>• YPAG meeting later in project than planned.<br>• No time to meet with co-researchers before YPAG meeting.<br>• Results summary & outstanding questions developed by RT & EG of McPin.<br>• Less time for review of circulated meeting materials.	• Co-researchers very successfully led the breakout rooms despite limited notice.<br>• Meeting with co-researchers to discuss YPAG feedback.<br>• Q&A session used as the basis for the final report discussion section and development of recommendations. GN & TS provided “final-eye” review to ensure perspectives of YPAG members interpreted correctly without researcher bias.
**Deliverables – lay summary**	• Written by co-researcher / another member of the YPAG.	• Delay in obtaining results & writing report = delay in briefing materials & less time to draft, review & make edits.<br>• Constrained by set template requiring some re-formatting / moving of sections / reducing word count of results section.	• Written by GN.
**Deliverables – infographic**	• Produced by co-researcher / another member of the YPAG.	• Delay in obtaining results & writing report = delay in briefing materials & less time to draft, review & make edits.<br>• Editable version sent to team lead to make suggested edits but no access to Adobe Illustrator – really tried to find same / similar images etc. but unfortunately had to use different programme & design; wording is the same but very sorry after all the effort put in for a great design.	• Drafted by another member of YPAG.

**Deliverables – 2-min video**	• Co-production with the co-researchers / other members of the YPAG.	• Requirement to send video script to funder start of Oct for sign-off before production by 3rd parties i.e., even without delay in obtaining results & writing report, no edits to the script and therefore the video could be made past this point.<br>• Decision was made for the video to be produced by team lead bypassing early sign-off by the funder allowing review & edits to be made right up to submission.<br>• Intended to have mock-up video to show in YPAG meeting but time spent finishing report.	• Video script reviewed by co-researchers, another member of the YPAG & RT of McPin.<br>• Video production was not completed by submission deadline, but this may provide an opportunity for the actual video to be reviewed by the co-researchers / YPAG as well.
**Further Opportunities**	• The team lead will stay in contact with the co-researchers over the coming year as there will likely be opportunities regarding sharing the results to the wider public.		

**Supplementary Table 3 - Searches**					
**Database Searches**					
**Database**	**Notes for Search Strings**	**Search**	**Date of Search**	**Search Results**	**Unique records after deduplication**
Pubmed	• Only AND, OR, NOT<br>• No proximity searching<br>• MeSH – some search terms missed out as PubMed searches for them anyway, including plurals / it duplicates the search<br>• * = truncation, stops automatic MeSH terms, best not to use unless specific term<br>• Anything out of “quotation” is automatically AND i.e., both terms present but anywhere	(depression OR depressive disorder, major OR MDD OR (depress* AND (disorder* OR "mood" OR symptom*)) OR (disorder* AND ("mood" OR "affective"))) AND (("young" AND ("people" OR person* OR adult* OR girl* OR "woman" OR "women" OR female* OR "boy" OR "boys" OR "man" OR "men" OR male*)) OR youth* OR (youth* AND ("global" OR "international")) OR adolescen* OR teen* OR student* OR "school aged" OR "school-aged") AND ("mobile health" OR "mhealth" OR "m-health" OR "electronic health" OR "ehealth" OR "e-health" OR "digital health" OR "electronic mental health" OR "e-mental health" OR "digital mental health" OR	16/08/2021	2123	2123

	in the text, “quotation” is exact phrase and significantly reduces search results<br>• No other wildcard characters	"telehealth" OR "telemedicine" OR "telepsychiatry" OR "digital phenotyping" OR technolog* OR mobile* OR "mobile phone*" OR smartphone* OR "cell phone*" OR "app" OR "apps" OR application* OR "smartphone-based" OR wearable* OR sensor* OR biosensor* OR bio-sensor* OR (("wearable" OR "electronic" OR "digital" OR "mobile") AND (device* OR diar*))) AND (((remote* OR continuous* OR "real-time" OR passive* OR electronic* OR "ambulatory") AND (measure* OR monitor* OR track* OR "sensing" OR assess* OR "feedback")) OR "ubiquitous sensing" OR "context sensing" OR "pervasive sensing" OR "personal sensing" OR "active sensing" OR "self-monitor*" OR "self-manage*" OR "mood track*" OR "activity track*" OR "ecological momentary assessment" OR "electronic momentary assessment" OR "experience sampling" OR "ecological momentary intervention*" OR "just-in-time intervention*" OR "personalised intervention*" OR "personalized intervention*")<br>1990 - 2021			
Ovid Embase 1974 – 2021 Week 32;<br>Global Health 1973 – 2021 Week 32;<br>APA PsychInfo 1806 – Aug 2021;<br>Ovid MEDLINE(R) ALL 1946 – Aug 2021	• AND, OR, NOT, ADJ<br>• * = truncation<br>• # = single letter<br>• ? = zero or single letter<br>• Automatic phrase searching / no need for quotation marks, but use of subheadings does reduce search results	depression/ OR depress* OR (depress* AND (disorder? OR symptom* OR mood)) OR "major depressive disorder" OR "MDD" OR (disorder? AND (mood OR affective))<br>limit 1 to yr="1990 -Current"<br>adolescent/ OR adolescen* OR (young AND (people OR person? OR adult? OR girl? OR wom#n OR female? OR boy? OR m#n OR male?)) OR youth? OR "global youth" OR teen* OR student? OR school-aged OR "school aged"<br>limit 3 to yr="1990 -Current"<br>telemedicine/ OR "mobile health" OR mhealth OR "m-health" OR "electronic health" OR ehealth OR "e-health" OR "digital health" OR "electronic mental health" OR "e-mental health" OR "digital mental health" OR telehealth OR	16/08/2021	2148	1096

		telemedicine OR telepsychiatry OR "digital phenotyping" OR technolog* OR mobile? OR "mobile phone?" OR smartphone? OR "cell phone?" OR "app" OR "apps" OR application? OR "smartphone-based" OR wearable? OR sensor? OR biosensor? OR bio-sensor? OR ((wearable OR electronic OR digital OR mobile) AND (device? OR diar*))<br>limit 5 to yr="1990 -Current"<br>monitoring/ OR ((remote* OR continuous* OR "real-time" OR passive* OR electronic* OR ambulatory) AND (measure* OR monitor* OR track* OR sensing OR assess* OR feedback)) OR "ubiquitous sensing" OR "context sensing" OR "pervasive sensing" OR "personal sensing" OR "active sensing" OR "self-monitor*" OR "self-manage*" OR "mood track*" OR "activity track*" OR "ecological momentary assessment" OR "electronic momentary assessment" OR "experience sampling" OR "ecological momentary intervention?" OR "just-in-time intervention?" OR "personali#ed intervention?"<br>limit 7 to yr="1990 -Current"<br>2 AND 4 AND 6 AND 8			
Web of Science<br>All Databases	• AND, OR, NOT, NEAR<br>• * = truncation<br>• ? = single letter<br>• $ = zero or single letter<br>• Anything out of "quotation" is automatically AND i.e. both terms present but anywhere in the text, "quotation" is exact phrase and significantly reduces search results	TS=(depress* OR (depress* AND (disorder$ OR symptom* OR mood)) OR "major depressive disorder" OR "MDD" OR (disorder$ AND (mood OR affective)))<br>1990-01-01 to 2021-08-16<br>TS=(adolescen* OR (young AND (people OR person$ OR adult$ OR girl$ OR wom?n OR female$ OR boy$ OR m?n OR male$)) OR youth$ OR "global youth" OR teen* OR student$ OR "school-aged" OR "school aged")<br>1990-01-01 to 2021-08-16<br>TS=("mobile health" OR mhealth OR "m-health" OR "electronic health" OR ehealth OR "e-health" OR "digital health" OR "electronic mental health" OR "e-mental health" OR "digital mental health"	16/08/2021	1758	1133

		OR telehealth OR telemedicine OR telepsychiatry OR "digital phenotyping" OR technolog* OR mobile$ OR "mobile phone$" OR smartphone$ OR "cell phone$" OR app OR apps OR application$ OR "smartphone-based" OR wearable$ OR sensor$ OR biosensor$ OR bio-sensor$ OR ((wearable OR electronic OR digital OR mobile) AND (device$ OR diar*)))<br>1990-01-01 to 2021-08-16<br>TS=(((remote* OR continuous* OR "real-time" OR passive* OR electronic* OR ambulatory) AND (measure* OR monitor* OR track* OR sensing OR assess* OR feedback)) OR "ubiquitous sensing" OR "context sensing" OR "pervasive sensing" OR "personal sensing" OR "active sensing" OR "self-monitor*" OR "self-manage*" OR "mood track*" OR "activity track*" OR "ecological momentary assessment" OR "electronic momentary assessment" OR "experience sampling" OR "ecological momentary intervention*" OR "just-in-time intervention*" OR "personali?ed intervention*")<br>1990-01-01 to 2021-08-16<br>#1 AND #2 AND #3 AND #4			
IEEE Xplore	• AND, OR, NOT, NEAR<br>• Limited to 20 search terms per search clause & 7 wild card characters total	((depress* OR ((depressive OR depression OR depressed) NEAR/2 (mood OR disorder OR symptom OR symptoms OR symptomatology)) OR "major depressive disorder" OR "MDD" OR ((disorder OR disorders) NEAR/2 (mood OR affective))) AND (adolescence OR adolescent OR adolescents OR (young NEAR/2 (people OR person OR persons OR adult OR adults OR girl OR girls OR woman OR women OR female OR females OR boy OR boys OR man OR men OR male OR males)) OR youth OR youths OR "global youth" OR teenager OR teenagers OR teen OR teens OR student OR "school-aged" OR "school aged") AND ("mobile health" OR mhealth OR "m-health" OR "electronic health"	16/08/2021	196	190

		OR ehealth OR "e-health" OR "digital health" OR "electronic mental health" OR "e-mental health" OR "digital mental health" OR telehealth OR telemedicine OR telepsychiatry OR "digital phenotyping" OR technology OR technologies OR mobile OR mobiles OR "mobile phone" OR "mobile phones" OR smartphone OR smartphones OR "cell phone" OR "cell phones" OR app OR apps OR application OR applications OR "smartphone-based" OR wearable OR wearables OR sensor OR sensors OR biosensor OR biosensors OR bio-sensor OR bio-sensors OR ((wearable OR electronic OR digital OR mobile) NEAR/2 (device OR devices OR diary OR diaries)) OR ((remote* OR continuous* OR "real-time" OR passive OR electronic OR ambulatory) NEAR/2 (measure* OR monitor* OR track* OR sensing OR assess* OR feedback)) OR "ubiquitous sensing" OR "context sensing" OR "pervasive sensing" OR "personal sensing" OR "active sensing" OR "self-monitor" OR "self-monitoring" OR "self-manage" OR "self-management" OR "mood tracker" OR "mood tracking" OR "activity tracker" OR "activity tracking" OR "ecological momentary assessment" OR "electronic momentary assessment" OR "experience sampling" OR "ecological momentary intervention" OR "just-in-time intervention" OR "personalised intervention" OR "personalized intervention"))<br>1990 – 2021			
HTA Database	• AND, OR, NOT<br>• * = truncation<br>• ? = single letter<br>• $ = zero or single letter<br>• Anything out of “quotation” is automatically AND i.e. both	(depress* OR (depress* AND (disorder$ OR symptom* OR mood)) OR "major depressive disorder" OR "MDD" OR (disorder$ AND (mood OR affective)))) AND (adolescen* OR (young AND (people OR person$ OR adult$ OR girl$ OR wom?n OR female$ OR boy$ OR m?n OR male$)) OR youth$ OR "global youth" OR teen*	16/08/2021	36	36

	terms present but anywhere in the text, “quotation” is exact phrase and significantly reduces search results	OR student$ OR "school-aged" OR "school aged") AND ("mobile health" OR mhealth OR "m-health" OR "electronic health" OR ehealth OR "e-health" OR "digital health" OR "electronic mental health" OR "e-mental health" OR "digital mental health" OR telehealth OR telemedicine OR telepsychiatry OR "digital phenotyping" OR technolog* OR mobile$ OR "mobile phone$" OR smartphone$ OR "cell phone$" OR app OR apps OR application$ OR "smartphone-based" OR wearable$ OR sensor$ OR biosensor$ OR bio-sensor$ OR ((wearable OR electronic OR digital OR mobile) AND (device$ OR diar*)) OR ((remote* OR continuous* OR "real-time" OR passive* OR electronic* OR ambulatory) AND (measure* OR monitor* OR track* OR sensing OR assess* OR feedback)) OR "ubiquitous sensing" OR "context sensing" OR "pervasive sensing" OR "personal sensing" OR "active sensing" OR "self-monitor*" OR "self-manage*" OR "mood track*" OR "activity track*" OR "ecological momentary assessment" OR "electronic momentary assessment" OR "experience sampling" OR "ecological momentary intervention*" OR "just-in-time intervention*" OR "personali?ed intervention*") FROM 1990 TO 2022			
ACM Digital Library	• Only OR, AND, NOT<br>• No proximity searching<br>• Assumes AND between searches, OR between search terms<br>• Specific phrase marked by quotation<br>• * = truncation<br>• ? = any single letter	Abstract:(depress* OR (depress* AND (disorder$ OR symptom* OR mood)) OR "major depressive disorder" OR "MDD" OR (disorder$ AND (mood OR affective))) AND Abstract:(adolescen* OR (young AND (people OR person$ OR adult$ OR girl$ OR wom?n OR female$ OR boy$ OR m?n OR male$)) OR youth$ OR "global youth" OR teen* OR student$ OR "school-aged" OR "school aged") AND Abstract:("mobile health" OR mhealth OR "m-health" OR "electronic health" OR ehealth OR "e-health" OR "digital health" OR "electronic	16/08/2021	227	226

	• Special characters need to be prefaced by / unless in quotation<br>• Wildcards cannot be used at the start of a search term or when searching for phrases within quotations	mental health" OR "e-mental health" OR "digital mental health" OR telehealth OR telemedicine OR telepsychiatry OR "digital phenotyping" OR technolog* OR mobile$ OR "mobile phone" OR "mobile phones" OR smartphone$ OR "cell phone" OR "cell phones" OR app OR apps OR application$ OR "smartphone-based" OR wearable$ OR sensor$ OR biosensor$ OR bio-sensor$ OR ((wearable OR electronic OR digital OR mobile) AND (device$ OR diar*)) OR ((remote* OR continuous* OR "real-time" OR passive* OR electronic* OR ambulatory) AND (measure* OR monitor* OR track* OR sensing OR assess* OR feedback)) OR "ubiquitous sensing" OR "context sensing" OR "pervasive sensing" OR "personal sensing" OR "active sensing" OR "self-monitor" OR "self-monitoring" OR "self-manage" OR "self-management" OR "mood tracker" OR "mood tracking" OR "activity tracker" OR "activity tracking" OR "ecological momentary assessment" OR "electronic momentary assessment" OR "experience sampling" OR "ecological momentary intervention" OR "just-in-time intervention" OR "personalised intervention" OR "personalized intervention")<br>Publication date Jan 1990 - Aug 2021			
Cochrane Library		Same as above, search each, send to search manager, #1 AND #2 AND #3 AND #4 Jan 1990 - Aug 2021	16/08/2021	446	317
			**Sub-total**	**6934**	**5121**

Grey Literature Searches

**Search Terms**	**Source**	**Search Results**
Depression<br>AND (young people OR youth OR adolescents)<br>AND technology	CADTH	**8**
	NICE	**93**
	WHO	**13**
	Clinicaltrials.gov	**110**
	ISRCTN registry	**8**

	MedRxiv	**562**
	Gov.uk	**171**
**Sub-total**		**965**
**Snowball Sampling, Hand Searching & Iterative Searches**		
**Search Terms**		**Search Results**
Search terms used in hand searching and iterative searches would depend on the theory being examined / new theories appearing during data extraction e.g. as above with the addition of “parent-report”, “school”, “data privacy”, “digital divide” etc**.**		**32**
**Total**		**6118**

**Supplementary Table 4 - Literature Selection, Appraisal & Data Extraction Form**<br>(originally conducted in excel with each table field a new column, and each record a new row)	
**Reference**<br>(exported from Mendeley Reference Manager)	
Source	Database, grey literature, hand/iterative searches
Authors	
Year	
Title	
Journal	
Volume	
Pages	
Keywords	
Abstract	
DOI	
**Literature Selection & Appraisal**	
Included / excluded?	
Reason for exclusion?	
Young person (14 – 25) specific?	
Type	Protocol, pilot/RCT, original research, review, case study, conference abstract
Main theory literature contains evidence for	**1. Intervention activities - what does & doesn’t work?**<br>*i. Methodological*<br>*ii. Real-time monitoring of variables relevant to MDD*<br>*iii. Clinical utility*<br>**2. Underlying mechanisms - how & why?**<br>**3. For whom?**

	i. Acceptability, continued engagement & factors affecting adherence<br>**4. In what context?**<br>*i. Implementation/integration into current care pathways*
Secondary theory literature contains evidence for	
Limitations section	
Relevance	
Rigour	
**Data Extraction**	
**Literature characteristics**	
Overall aim	
Country	
Context	E.g., community-based, school/university, clinic (primary/secondary care), RCT
**Participant characteristics**	
Population	E.g., community sample, school/university students, high-risk, sub-threshold symptoms, clinical diagnosis of depression, internalising symptoms
Sample size (after exclusions)	
Age	
Presence of depression?	
Other characteristics	E.g., sociodemographic, physical/mental health comorbidities
**Intervention activities - what does & doesn't work?**	
Methodological	
Type of RMT used	Smartphone, smartphone apps, wearable, internet-based dashboard that can be accessed on portable device
Name	
Passive and/or active monitoring?	
Sensors used & data collected	
Sampling frequency & length of data collection	
Other	E.g., study or own smartphone, operating system (Android or iPhone), data processing
Real-time monitoring of variables relevant to MDD	
Ground-truth/validation measure of depression	Clinical interview, questionnaire measures & cut-offs etc.
Accuracy as a proxy for depression symptom severity (classification, association, prediction)	Details of analysis, effect sizes etc.
Sensitivity & specificity for depression	Details of analysis, effect sizes etc.
Potential other factors influencing accuracy	E.g., inclusion of parent report
Clinical Utility	
Use	Screening, symptom management, relapse-prevention, personalised intervention strategies
Feedback type	Feedback to patient for self-monitoring, feedback to HPC, data used for EMIs/JITAIs

Affect on depression outcome measures	Details of analysis, effect sizes etc.
Other outcome measures?	
Unintended outcomes?	
**Underlying mechanisms - how & why?**	
Do the authors measure or propose any mechanisms for intended outcomes?	E.g., objectivity, ecological validity, less recall/mood-state bias? Quicker response? Inform/shared clinical decision-making? Improved patient-clinician communication? Improved insight, self-awareness? Encourage goal setting, behaviour change, help-seeking? Monitor effects of other interventions?
Do the authors measure or propose any mechanisms for unintended outcomes?	E.g., false positives/negatives, misinterpretation? Relapse-signature but missed/ignored/waiting lists? Overfocusing on symptoms and health anxiety? Daily confrontation of perceived shortcomings? Non-adherence/technological issues, data loss & inconsistent monitoring? Reduced face-to-face interaction and overall quality of care?
**For Whom?**	
Acceptability, continued engagement & factors affecting adherence	
Measures of acceptability	E.g., usage statistics, drop-out over time, EMA prompt response rates
Health-related	E.g., symptom severity, motivation, comorbidities
User-related	E.g., age, technology acceptance, digital literacy, data privacy, overall value (perceived utility vs. costs)
Technology-related	E.g., useability, customizability, convenience, accessibility, accuracy vs. intrusiveness
Conflicting views of young people and HCPs and managing expectations	E.g., changes in roles and responsibilities of HCPs, expectation of quicker response
**In what context?**	
Implementation / integration into current care pathways	
IT Infrastructure	E.g., alert-based systems, link to electronic health records
Use in schools/universities	
Digital divides	
LMICs	
**Snowball sampling / hand searches**	
Reference	
