# Supplementary material for: A collaborative realist review of remote measurement technologies for depression in young people
Source: Nat Hum Behav. 2024 Jan 15;8(3):480–92. doi: 10.1038/s41562-023-01793-5 (PMC10963268; doi:10.1038/s41562-023-01793-5)
Supplement: Supplementary file 2 — Reporting Summary [file 41562_2023_1793_MOESM2_ESM.pdf]

nature portfolio

Corresponding author(s): Annabel Walsh

Last updated by author(s): 10/11/2023

Reporting Summary

Nature Portfolio wishes to improve the reproducibility of the work that we publish. This form provides structure for consistency and transparency in reporting. For further information on Nature Portfolio policies, see our Editorial Policies and the Editorial Policy Checklist.

Statistics

For all statistical analyses, confirm that the following items are present in the figure legend, table legend, main text, or Methods section.

n/a	Confirmed	
☐	☒	The exact sample size (*n*) for each experimental group/condition, given as a discrete number and unit of measurement
☒	☐	A statement on whether measurements were taken from distinct samples or whether the same sample was measured repeatedly
☒	☐	The statistical test(s) used AND whether they are one- or two-sided<br>*Only common tests should be described solely by name; describe more complex techniques in the Methods section.*
☒	☐	A description of all covariates tested
☒	☐	A description of any assumptions or corrections, such as tests of normality and adjustment for multiple comparisons
☐	☒	A full description of the statistical parameters including central tendency (e.g. means) or other basic estimates (e.g. regression coefficient) AND variation (e.g. standard deviation) or associated estimates of uncertainty (e.g. confidence intervals)
☒	☐	For null hypothesis testing, the test statistic (e.g. *F*, *t*, *r*) with confidence intervals, effect sizes, degrees of freedom and *P* value noted<br>*Give P values as exact values whenever suitable.*
☒	☐	For Bayesian analysis, information on the choice of priors and Markov chain Monte Carlo settings
☒	☐	For hierarchical and complex designs, identification of the appropriate level for tests and full reporting of outcomes
☒	☐	Estimates of effect sizes (e.g. Cohen's *d*, Pearson's *r*), indicating how they were calculated

Our web collection on statistics for biologists contains articles on many of the points above.

Software and code

Policy information about availability of computer code

Data collection	Clarivate EndNote 20<br>Microsoft Office 365 Excel
Data analysis	Microsoft Office 365 Excel

For manuscripts utilizing custom algorithms or software that are central to the research but not yet described in published literature, software must be made available to editors and reviewers. We strongly encourage code deposition in a community repository (e.g. GitHub). See the Nature Portfolio guidelines for submitting code & software for further information.

Data

Policy information about availability of data

All manuscripts must include a data availability statement. This statement should provide the following information, where applicable:

- Accession codes, unique identifiers, or web links for publicly available datasets
- A description of any restrictions on data availability
- For clinical datasets or third party data, please ensure that the statement adheres to our policy

This manuscript comprises a realist review, for which a variety of databases were searched for relevant literature, from which data was extracted. The databases used are listed below, and all included literature has been referenced, but data availability may be limited depending on whether or not the database/literature is open access. Web links are provided for publicly available databases.

PubMed – https://pubmed.ncbi.nlm.nih.gov/; Ovid (EMBASE, MEDLINE, APA PsychINFO and Global Health); Web of Science; Cochrane Library – https://www.cochranelibrary.com/; IEEE Xplore – https://ieeexplore.ieee.org/Xplore/home.jsp; HTA database – https://database.inahta.org/; ACM digital library – https://dl.acm.org/; CADTH – https://www.cadth.ca/; NICE – https://www.nice.org.uk/; WHO - https://www.who.int/; ClinicalTrails.gov – https://clinicaltrials.gov/; ISRCTN registry – https://www.isrctn.com/; Gov.uk – https://www.gov.uk/; arXiv – https://arxiv.org/.

Research involving human participants, their data, or biological material

Policy information about studies with human participants or human data. See also policy information about sex, gender (identity/presentation), and sexual orientation and race, ethnicity and racism.

Reporting on sex and gender	N/A
Reporting on race, ethnicity, or other socially relevant groupings	N/A
Population characteristics	Not research participants, rather lived experience involvement via consultation with the McPin Young People's Advisory Group (YPAG) (14 members, aged 14 - 25), and 2 young people co-researchers (1 male, 1 female) with lived experience of past history and/or current depression. Whilst demographic data is considered upon recruitment to the YPAG/involvement opportunities to ensure diversity of voices, we do not store this data and therefore cannot include it here.
Recruitment	Recruited through The McPin Foundation
Ethics oversight	The McPin Foundation

Note that full information on the approval of the study protocol must also be provided in the manuscript.

Field-specific reporting

Please select the one below that is the best fit for your research. If you are not sure, read the appropriate sections before making your selection.

☐ Life sciences ☒ Behavioural & social sciences ☐ Ecological, evolutionary & environmental sciences

For a reference copy of the document with all sections, see nature.com/documents/nr-reporting-summary-flat.pdf

Behavioural & social sciences study design

All studies must disclose on these points even when the disclosure is negative.

Study description	Realist review of qualitative, quantitative, and mixed-methods study designs, as well as conference proceedings, protocols, and other gray literature.
Research sample	Previously published literature relevant to the use of remote measurement technologies for depression in young people aged 14 - 24 years.
Sampling strategy	Exploratory searches, followed by iterative purposive searches, as well as snowball sampling and hand searching references. Searches were as comprehensive as possible, but the amount of evidence available for synthesis limited by the amount of relevant previously published literature available.
Data collection	A bespoke data extraction form was created in Excel by AW, with evidence extracted from relevant previously published literature and inputted into the form by AW, GN, TS, ZZ & MM. No researchers were blind to the study hypothesis.
Timing	Searchers were conducted in August 2021.
Data exclusions	Literature excluded n = 5703. Literature where remote measurement technologies were used solely to deliver an intervention without any remote measurement, focused on a specific type of depression (e.g., bipolar, perinatal, or postnatal), or did not include a standardized measure of depression (e.g., well-being scales) was excluded.
Non-participation	Not applicable - the study was a realist review of relevant previously published literature, with no human participants involved in the study.
Randomization	Not applicable - the study was a realist review of relevant previously published literature, with no human participants involved in the study.

Reporting for specific materials, systems and methods

We require information from authors about some types of materials, experimental systems and methods used in many studies. Here, indicate whether each material, system or method listed is relevant to your study. If you are not sure if a list item applies to your research, read the appropriate section before selecting a response.

Materials & experimental systems

n/a	Involved in the study
☒	☐ Antibodies
☒	☐ Eukaryotic cell lines
☒	☐ Palaeontology and archaeology
☒	☐ Animals and other organisms
☒	☐ Clinical data
☒	☐ Dual use research of concern
☒	☐ Plants

Methods

n/a	Involved in the study
☒	☐ ChIP-seq
☒	☐ Flow cytometry
☒	☐ MRI-based neuroimaging
